# Diagnostic Accuracy of the *Leishmania* OligoC-TesT and NASBA-Oligochromatography for Diagnosis of Leishmaniasis in Sudan

**DOI:** 10.1371/journal.pntd.0000776

**Published:** 2010-08-03

**Authors:** Alfarazdeg A. Saad, Nuha G. Ahmed, Osman S. Osman, Ahmed Almustafa Al-Basheer, Awad Hamad, Stijn Deborggraeve, Philippe Büscher, Gerard J. Schoone, Henk D. Schallig, Thierry Laurent, Ahmed Haleem, Omran F. Osman, Ahmed Mohamedain Eltom, Mustafa I. Elbashir, Sayda El-Safi

**Affiliations:** 1 Department of Biochemistry, Khartoum University, Sudan; 2 Department of Microbiology and Parasitology, Khartoum University, Sudan; 3 Department of Parasitology, Institute of Tropical Medicine, Antwerp, Belgium; 4 Rega Institute, Katholieke Universiteit Leuven, Leuven, Belgium; 5 KIT Biomedical Research, Parasitology Unit, Koninklijk Instituut voor de Tropen (KIT), Amsterdam, The Netherlands; 6 Coris BioConcept, Gembloux, Belgium; 7 Faculty of Laboratory Science, Khartoum University, Sudan; 8 Faculty of Science, Khartoum University, Sudan; Lancaster University, United Kingdom

## Abstract

**Background:**

The *Leishmania* OligoC-TesT and NASBA-Oligochromatography (OC) were recently developed for simplified and standardised molecular detection of *Leishmania* parasites in clinical specimens. We here present the phase II evaluation of both tests for diagnosis of visceral leishmaniasis (VL), cutaneous leishmaniasis (CL) and post kala-azar dermal leishmaniasis (PKDL) in Sudan.

**Methodology:**

The diagnostic accuracy of the tests was evaluated on 90 confirmed and 90 suspected VL cases, 7 confirmed and 8 suspected CL cases, 2 confirmed PKDL cases and 50 healthy endemic controls from Gedarif state and Khartoum state in Sudan.

**Principal Findings:**

The OligoC-TesT as well as the NASBA-OC showed a sensitivity of 96.8% (95% CI: 83.8%–99.4%) on lymph node aspirates and of 96.2% (95% CI: 89.4%–98.7%) on blood from the confirmed VL cases. The sensitivity on bone marrow was 96.9% (95% CI: 89.3%–99.1%) and 95.3% (95% CI: 87.1%–98.4%) for the OligoC-TesT and NASBA-OC, respectively. All confirmed CL and PKDL cases were positive with both tests. On the suspected VL cases, we observed a positive OligoC-TesT and NASBA-OC result in 37.1% (95% CI: 23.2%–53.7%) and 34.3% (95% CI: 20.8%–50.9%) on lymph, in 72.7% (95% CI: 55.8%–84.9%) and 63.6% (95% CI: 46.6%–77.8%) on bone marrow and in 76.9% (95% CI: 49.7%–91.8%) and 69.2% (95% CI: 42.4%–87.3%) on blood. Seven out of 8 CL suspected cases were positive with both tests. The specificity on the healthy endemic controls was 90% (95% CI: 78.6%–95.7%) for the OligoC-TesT and 100% (95% CI: 92.9%–100.0%) for the NASBA-OC test.

**Conclusions:**

Both tests showed high sensitivity on lymph, blood and tissue scrapings for diagnosis of VL, CL and PKDL in Sudan, but the specificity for clinical VL was significantly higher with NASBA-OC.

## Author Summary

The leishmaniases are a group of vector-borne diseases caused by protozoan parasites of the genus *Leishmania*. The parasites are transmitted by phlebotomine sand flies and can cause, depending on the infecting species, three clinical manifestations of leishmaniasis: visceral leishmaniasis (VL), post kala-azar dermal leishmaniasis (PKDL) and cutaneous leishmaniasis (CL) including the mucocutaneous form. VL, PKDL as well as CL are endemic in several parts of Sudan, and VL especially represents a major health problem in this country. Molecular tests such as the polymerase chain reaction (PCR) or nucleic acid sequence based assay (NASBA) are powerful techniques for accurate detection of the parasite in clinical specimens, but broad use is hampered by their complexity and lack of standardisation. Recently, the *Leishmania* OligoC-TesT and NASBA-Oligochromatography were developed as simplified and standardised PCR and NASBA formats. In this study, both tests were phase II evaluated for diagnosis of VL, PKDL and CL in Sudan.

## Introduction

The leishmaniases are a group of vector-borne diseases caused by parasites of the genus *Leishmania*. The parasites are transmitted by phlebotomine sand flies and can cause, depending on the infecting species, three main clinical manifestations of leishmaniasis: visceral leishmaniasis (VL), post kala-azar dermal leishmaniasis (PKDL) and cutaneous leishmaniasis (CL) including the mucocutaneous form [1]. VL is the most severe form in which the internal organs are invaded and tends to be 100% fatal if not appropriately treated. While CL and MCL are clinical manifestations as a result from replication of the parasite in the dermis and naso-oropharyngeal mucosa respectively, PKDL is a skin disorder seen in a number of treated VL patients. VL, PKDL and CL are endemic in several parts of Sudan and especially VL represents a major health problem in this country [2].

Although serological tests such as the direct agglutination test (DAT) [3], [4] and the rK39 dipstick test [5]–[7] have become the mainstay in VL diagnosis [8], parasite detection by microscopic analysis of aspirates from the lymph nodes, bone marrow or spleen is still used in some endemic regions. The diagnostic standard for CL and PKDL is microscopic analysis of tissue biopsies or scrapings. However, microscopy is hampered by its low and variable sensitivity and the need for rather invasive sampling in the case of VL. Sensitivity may be increased by prior *in vitro* cultivation of the parasite, but this technique is cumbersome and time consuming. Serological tests are of high value to support clinical diagnosis of VL but they are less useful in patients co-infected with HIV [9] and antibodies remain detectable for years after successful treatment [10], [11]. Antibody detection with the rapid rK39 dipstick test is not yet implemented in Sudan due to the reported low diagnostic performance of the test in this region [12]–[14].

Molecular diagnostics such as the polymerase chain reaction (PCR) and nucleic acid sequence based assay (NASBA) offer attractive alternatives to conventional parasite detection as they are generally highly sensitive and specific. PCR detects the parasite's DNA through *in vitro* thermocyclic conditions [15], while NASBA amplifies the RNA by an isothermal reaction [16]. However, broad application of these powerful techniques in diagnosis and control of leishmaniasis is hindered by their complexity and lack of standardised test formats. Recently, the *Leishmania* OligoC-TesT and NASBA-Oligochromatography (OC) were introduced as promising PCR and NASBA formats for simplified and standardised molecular detection of *Leishmania* parasites [17], [18]. The tests are based on amplification of a short sequence within the *Leishmania* 18S ribosomal DNA (PCR) or RNA (NASBA), followed by simple and rapid detection of amplified products in dipstick format. Both tests are available as self-containing kits including all components needed except for the *Taq* polymerase enzyme in the OligoC-TesT and the Nuclisense Basic Kit in the NASBA-OC due to licensing issues. The tests showed high sensitivity and specificity on experimentally prepared specimens and on clinical specimens from a limited number of confirmed cases and healthy controls (phase I) [17], [18] and satisfactory repeatability and reproducibility in a multicenter evaluation study [19]. In this phase II study, we evaluated the two tests in Sudan on confirmed and suspected VL, CL and PKDL patients and on healthy endemic controls.

## Methods

### Participants

VL and PKDL suspects and endemic healthy controls were recruited in Gedarif State and CL suspects in Khartoum State between October 2008 and January 2009. VL and PKDL suspects were recruited in the health centers of villages within the endemic areas while the endemic healthy controls were volunteers from the same villages but not visiting the health centers. CL suspects were recruited at the Dermatology Hospital in Khartoum. Confirmed leishmaniasis cases were given appropriate treatment in the same health center or hospital as they were diagnosed. A participant was included in the study if 2 years or older and written consent was obtained from the individual or his/her guardian. Specimen collection was performed by the Faculty of Medicine of Khartoum University. Ethical clearance for the study was obtained from the ethical committees of the Federal Ministry of Health Committee in Sudan and the University of Antwerp in Belgium.

### Participant classification and reference tests

A participant was classified as (i) confirmed VL case if there was clinical suspicion for VL, DAT on serum was positive (titer ≥1∶3200) and parasites were observed in lymph or bone marrow aspirates by microscopic analysis; (ii) suspected VL case with positive DAT if there was clinical suspicion for VL, DAT on serum was positive, no parasites were observed in lymph or bone marrow aspirates and no previous history of VL was reported; (iii) suspected VL case with negative DAT if there was clinical suspicion for VL, DAT on serum was negative, no parasites were observed in lymph or bone marrow aspirates and no previous history of VL was reported; (iv) endemic healthy control if there was no clinical suspicion for VL, no previous history of VL and DAT on serum was negative; (v) confirmed CL or PDKL case if there was clinical suspicion for CL or PKDL and parasites were observed in lesion or skin scrapings by microscopic analysis; and (vi) suspected CL case if there was clinical suspicion for CL and no parasites were observed in lesion or skin scrapings. Clinical suspicion for VL was defined as a history of fever for two weeks or more and splenomegaly or lymphadenopathy and for CL and PKDL the presence of skin lesions or nodules. The reference tests were performed by experienced laboratory technicians immediately after specimen taking at the collection sites as described in the WHO manual on visceral leishmaniasis [20].

### Index tests

Two hundred µl anti-coagulated blood (confirmed and suspected VL cases and healthy endemic controls), and/or inguinal lymph node aspirate, and/or bone marrow aspirate (confirmed and suspected VL cases) or lesion or skin scrapings (confirmed and suspected CL and PKDL cases) was mixed with 200 µl of AngeroNA buffer (Mallinckrodt Baker, USA). This buffer allows specimen storage without loss of DNA and RNA quality. The same specimens were used for the reference tests and for the index tests. Specimens were shipped at 4°C from the collection site to Soba University hospital laboratory of Khartoum University and stored at 4°C for a maximum of two weeks. Nucleic acids of the specimens were extracted according to the method described by Boom *et al.* [21]. Elution was done in 50 µl of Tris-EDTA (TE) buffer and stored at −20°C until analysis. The extracts were tested with the *Leishmania* OligoC-TesT and NASBA-OC between October 2008 and February 2009 as described by Deborggraeve et al. [17] and Mugasa et al. [18]. In brief, with the OligoC-TesT DNA of the parasite is amplified by PCR and subsequently 40 µl of denatured PCR product is mixed with 40 µl of migration buffer preheated at 55°C for at least 20 minutes followed by dipping the Oligo-strip into the solution. The NASBA-OC amplifies an RNA sequence of the parasite by NASBA reaction after which 4 µl of the amplified product is mixed with 76 µl of migration buffer preheated at 55°C and the Oligo-strip is dipped into the solution. Test results are read after 10 minutes for both tests. Executors of the index tests were trained during a one-week workshop held in June 2007 at Makerere University, Kampala, Uganda. No external quality control confirming the reference test or index test results could be performed during the study. Executors of the index tests were not blinded to the results of the reference tests.

### Data analysis

The sensitivity and specificity of the *Leishmania* OligoC-TesT and NASBA-OC were calculated from data entered into contingency tables. Differences in sensitivity and specificity between the two tests and differences in test results of specimen types were estimated by the Mc Nemar test. Concordances between the two tests were determined using the kappa index. All calculations were estimated at a 95% confidence interval (95% CI).

## Results

### Participants

In the study the following participants were recruited: 90 confirmed and 67 suspected VL cases with positive DAT, 23 suspected VL cases with negative DAT, 7 confirmed and 8 suspected CL cases, 2 confirmed PKDL cases and 50 healthy endemic controls.

### Sensitivity and specificity of the Leishmania OligoC-TesT and NASBA-OC

#### Confirmed VL, CL and PKDL patients

The *Leishmania* OligoC-TesT and the NASBA-OC showed both a sensitivity of 96.8% on lymph node aspirates from confirmed VL cases and of 96.2% on blood (Table 1). The sensitivity on bone marrow was 96.9% and 95.3% for the OligoC-TesT and NASBA-OC, respectively. All confirmed CL (n = 7) and PKDL (n = 2) cases were positive with the OligoC-TesT and NASBA-OC. There was no significant difference in sensitivity between the two index tests (*P*>0.05). When analysing different specimen types from the same VL patient we observed no significant difference in test outcome (*P*>0.05).

**Table 1:** Diagnostic accuracy of the *Leishmania* OligoC-TesT and NASBA-OC on confirmed and suspected leishmaniasis cases and on healthy endemic controls from Sudan.

Participants	Number of specimens	OligoC-TesT			NASBA-OC		
		Number of positives	Sensitivity % (95% CI)	Specificity % (95% CI)	Number of positives	Sensitivity % (95% CI)	Specificity % (95% CI)
Confirmed cases							
VL							
Lymph	31	30	96.8 (83.8–99.4)		30	96.8 (83.8–99.4)	
Blood	79	76	96.2 (89.4–98.7)		76	96.2 (89.4–98.7)	
Bone marrow	64	62	96.9 (89.3–99.1)		61	95.3 (87.1–98.4)	
CL							
Lesion scrapings	7	7	100 (64.6–100)		7	100 (64.6–100)	
PKDL							
Skin scrapings	2	2	100 (34.2–100)		2	100 (34.2–100)	
Suspected cases							
Suspected VL with pos DAT							
Lymph	35	13	37.1 (23.2–53.7)		12	34.3 (20.8–50.9)	
Blood	13	10	76.9 (49.7–91.8)		9	69.2 (42.4–87.3)	
Bone marrow	33	24	72.7 (55.8–84.9)		21	63.6 (46.6–77.8)	
Suspected VL with neg DAT							
Lymph	10	3	30 (10.8–60.3)		3	30 (10.8–60.3)	
Blood	14	4	28.6 (11.7–54.7)		6	42.9 (24.4–67.4)	
Bone marrow	14	6	42.9 (24.4–67.4)		7	50 (26.8–73.2)	
CL							
Lesion scrapings	8	7	87.5 (52.9–97.8)		7	87.5 (52.9–97.8)	
Healthy endemic controls							
Blood	50	5		90 (78.6–95.7)	0		100 (92.9–100)

Note. CI = confidence interval, VL = visceral leishmaniasis, CL = cutaneous leishmaniasis, PKDL = post kala azar dermal leishmaniasis, pos = positive, neg = negative, DAT = direct agglutination test.

#### Suspected VL and CL patients

On the suspected VL cases with positive DAT, the OligoC-TesT showed a positive test result in 37.1%, 76.9% and 72.7% on lymph, blood and bone marrow, respectively; while for NASBA-OC this was 34.3%, 69.2% and 63.6% (Table 1). On the suspected VL cases with negative DAT, the OligoC-TesT was positive in 30%, 28.6% and 42.9% on lymph, blood and bone marrow and the NASBA-OC in 30%, 42.9% and 50%. On the 8 CL suspected cases 7 were positive with the OligoC-TesT and the NASBA-OC. The Mc Nemar test indicated no significant difference in results of the two tests on the specimens from suspected cases (*P*>0.05). We observed no significant difference in test outcome when different specimen types from the same suspected VL patient were analysed (*P*>0.05).

#### Healthy endemic controls

Five of the 50 healthy endemic controls showed a positive OligoC-TesT result, while the NASBA-OC remained negative on all endemic controls (Table 1). Hence, the specificity of the OligoC-TesT for disease was 90.0% and of the NASBA-OC 100.0%, which is significantly higher (*P*<0.05).

#### Test agreement

On all specimens analysed (n = 321) the two tests showed a kappa value of 0.85 (95% CI: 0.75–0.96) indicating almost perfect agreement [22]. An overview of the agreement analysis on the different specimen types is presented in table 2.

**Table 2:** Agreement between the *Leishmania* OligoC-TesT and NASBA-OC on blood, lymph, bone marrow and lesion scraping specimens.

Specimen type	Number of specimens	Number of specimens with agreement	Kappa value (95% CI)
Blood	142	134	0.89 (0.72–1)
Lymph	66	59	0.77 (0.53–1)
Bone marrow	97	93	0.82 (0.63–1)
Lesion scrapings	17	17	1 (0.53–1.48)
Total	321	302	0.85 (0.75–0.96)

## Discussion

Molecular diagnostics are attractive alternatives to conventional parasite detection as they combine sensitivity with specificity. Recently, the *Leishmania* OligoC-TesT and NASBA-OC were developed as standardised formats for molecular detection of *Leishmania* parasites [17], [18]. Here we present the phase II evaluation of the two tests on confirmed and suspected VL, CL and PKDL cases and on health endemic controls from Sudan. Although the study was set out as a phase II diagnostic evaluation, it has some limitations. A weak point is the lack of external quality control on a subset of specimens carried out by another reference laboratory. Furthermore, the executors of the index tests were not blinded to the participant classification and thus the results of the reference tests. Although we are convinced that these limitations did not influence the study results, these shortcomings should be avoided in future phase II and III evaluation trials. In addition, response to treatment was not included in the standard references as we were not able to follow up the patients after treatment. Lastly, the number of participants in some subgroups is too low to make major conclusions.

The *Leishmania* OligoC-TesT as well as the NASBA-OC showed a high sensitivity (>95%) on lymph, blood and bone marrow from the confirmed VL patients. The observation that analysing lymph or blood yielded similar sensitivity as bone marrow is very promising towards less invasive diagnosis. Indeed this means that the OligoC-TesT and NASBA-OC on lymph or blood could indicate the infection status of VL suspected cases. Similar findings were reported in a study on the phase III evaluation of conventional PCR for VL diagnosis in Nepal [23]. Although the number of confirmed CL and PKDL cases in our study was limited, all were positive with both tests indicating their potential for accurate detection of the parasites in skin tissues. In addition, the high sensitivity is confirmed by the observation that the tests were able to detect *Leishmania* RNA or DNA in the blood or lesion scrapings of to the majority of the suspected VL and CL cases. Several cases in the suspected patient with positive DAT group might be true leishmaniasis cases given the suboptimal sensitivity of the conventional parasite detection tests. In 2008, Deborggraeve et al. reported a sensitivity of conventional PCR on probable VL cases (defined as clinical suspicion of VL and positive DAT test but negative in conventional parasite detection) of 67.6% on blood and 71.8% on bone marrow [23]. The sensitivities of the index tests on the lymph node aspirates of the suspected VL cases is surprisingly low (34–37%). This might indicate that the parasite load in blood is higher than in lymph. The fact that this is not observed in the confirmed VL cases is probably due to the general higher parasite load in this patient group because of the low sensitivity of the reference test. PCR positivity in the suspected VL group with negative DAT was not significantly lower than with positive DAT. This confirms the low correlation in DAT and PCR status in these groups as observed earlier [23]. While antibody levels in the blood are a marker for the host response, the PCR/NASBA status of the blood is a marker for the presence of parasites and might therefore be complementary. PCR and NASBA can offer an added value compared to immunodiagnosis in HIV co-infected VL patients.

On the 50 healthy endemic controls, the OligoC-TesT showed a specificity of 90% while this was 100% for the NASBA-OC. The positive OligoC-TesT results might be due to asymptomatic infections, which are known to be common in VL endemic regions. The inclusion of these asymptomatic carriers in the control group could be explained by the low concordance between negative DAT status and PCR outcome on blood from endemic control persons as described by Deborggraeve et al. [23] and Bhattarai et al. [24]. However, while NASBA-OC showed equal sensitivity on the confirmed and suspected VL cases, the test did not detect these assumed asymptomatic infections in the endemic control group. This discrepancy can be explained in two ways. Firstly, although not indicated by the negative controls taken along in specimen analysis, contamination of the PCR can never be fully excluded. Secondly, the OligoC-TesT might be slightly more sensitive than the NASBA-OC and thus pick up asymptomatic infections which might have very low parasite loads in the blood. The observed equal sensitivity for both tests on the confirmed and suspected VL cases might be biased by the fact that these cases are individuals presenting syndromes and thus probably have higher parasite loads than healthy parasite carriers. Hence, NASBA-OC might provide a better marker for active disease than the OligoC-TesT, as NASBA-OC does not detect asymptomatic infections while more than 95% of the active VL cases are still positive with the test. Furthermore, both tests might be useful as a test of cure after treatment of VL. As cure does not always equals parasite clearance, NASBA-OC might be more suitable than the OligoC-TesT. However, specific evaluation studies are needed to confirm this hypothesis.

One should keep in mind that the PCR-OC and NASBA-OC are not yet an option for routine diagnosis at the primary care level as they require basic molecular biology lab facilities. VL typically affects populations in rural areas where health centers most often suffer from infrastructural limitations and thus only apply less sophisticated diagnostic methods. Yet, they can be valuable tools in leishmaniasis diagnosis at reference hospitals with basic molecular biology lab facilities. In addition, the evaluated tools can offer an added value in disease surveillance and epidemiological studies in which specimens are analysed at central reference laboratories.

In conclusion, the *Leishmania* OligoC-TesT and NASBA-OC showed high sensitivity on lymph and blood of VL patients and on scrapings from CL and PKDL patients from Sudan. A significant higher specificity for active VL with the NASBA-OC than with the OligoC-TesT was observed. Both tests are not yet an option for routine diagnosis of leishmaniasis at the primary care level due to their infrastructural requirements but they might be powerful diagnostic tests in disease surveillance programmes and in monitoring intervention studies.

## Supporting Information

Checklist S1 STARD Checklist
(0.13 MB PDF)
