# Supplementary material for: Diagnostic Accuracy of the Leishmania OligoC-TesT and NASBA-Oligochromatography for Diagnosis of Leishmaniasis in Sudan
Source: PLoS Negl Trop Dis. 2010 Aug 3;4(8):e776. doi: 10.1371/journal.pntd.0000776 (PMC2914782; doi:10.1371/journal.pntd.0000776)
Supplement: Checklist S1 — STARD Checklist (0.13 MB PDF) [file pntd.0000776.s001.pdf]

Table 1. STARD checklist for the reporting of studies of diagnostic accuracy.

Section and Topic	Item #		On page #
TITLE/ABSTRACT/ KEYWORDS	1	Identify the article as a study of diagnostic accuracy (recommend MeSH heading 'sensitivity and specificity').	1
INTRODUCTION	2	State the research questions or study aims, such as estimating diagnostic accuracy or comparing accuracy between tests or across participant groups.	7
METHODS		Describe	
Participants	3	The study population: The inclusion and exclusion criteria, setting and locations where the data were collected.	8
	4	Participant recruitment: Was recruitment based on presenting symptoms, results from previous tests, or the fact that the participants had received the index tests or the reference standard?	8
	5	Participant sampling: Was the study population a consecutive series of participants defined by the selection criteria in items 3 and 4? If not, specify how participants were further selected.	8
	6	Data collection: Was data collection planned before the index test and reference standard were performed (prospective study) or after (retrospective study)?	8
Test methods	7	The reference standard and its rationale.	
	8	Technical specifications of material and methods involved including how and when measurements were taken, and/or cite references for index tests and reference standard.	8 - 9
	9	Definition of and rationale for the units, cutoffs and/or categories of the results of the index tests and the reference standard.	8 - 9
	10	The number, training and expertise of the persons executing and reading the index tests and the reference standard.	8 - 9
	11	Whether or not the readers of the index tests and reference standard were blind (masked) to the results of the other test and describe any other clinical information available to the readers.	10
Statistical methods	12	Methods for calculating or comparing measures of diagnostic accuracy, and the statistical methods used to quantify uncertainty (e.g. 95% confidence intervals).	10
	13	Methods for calculating test reproducibility, if done.	NA
RESULTS		Report	
Participants	14	When study was done, including beginning and ending dates of recruitment.	8
	15	Clinical and demographic characteristics of the study population (e.g. age, sex, spectrum of presenting symptoms, comorbidity, current treatments, recruitment centers).	NA
	16	The number of participants satisfying the criteria for inclusion that did or did not undergo the index tests and/or the reference standard; describe why participants failed to receive either test (a flow diagram is strongly recommended).	11
Test results	17	Time interval from the index tests to the reference standard, and any treatment administered between.	8 - 9
	18	Distribution of severity of disease (define criteria) in those with the target condition; other diagnoses in participants without the target condition.	11
	19	A cross tabulation of the results of the index tests (including indeterminate and missing results) by the results of the reference standard; for continuous results, the distribution of the test results by the results of the reference standard.	20
	20	Any adverse events from performing the index tests or the reference standard.	NA
Estimates	21	Estimates of diagnostic accuracy and measures of statistical uncertainty (e.g. 95% confidence intervals).	11 - 12
	22	How indeterminate results, missing responses and outliers of the index tests were handled.	NA
	23	Estimates of variability of diagnostic accuracy between subgroups of participants, readers or centers, if done.	11 - 12
	24	Estimates of test reproducibility, if done.	NA
DISCUSSION	25	Discuss the clinical applicability of the study findings.	13 - 15

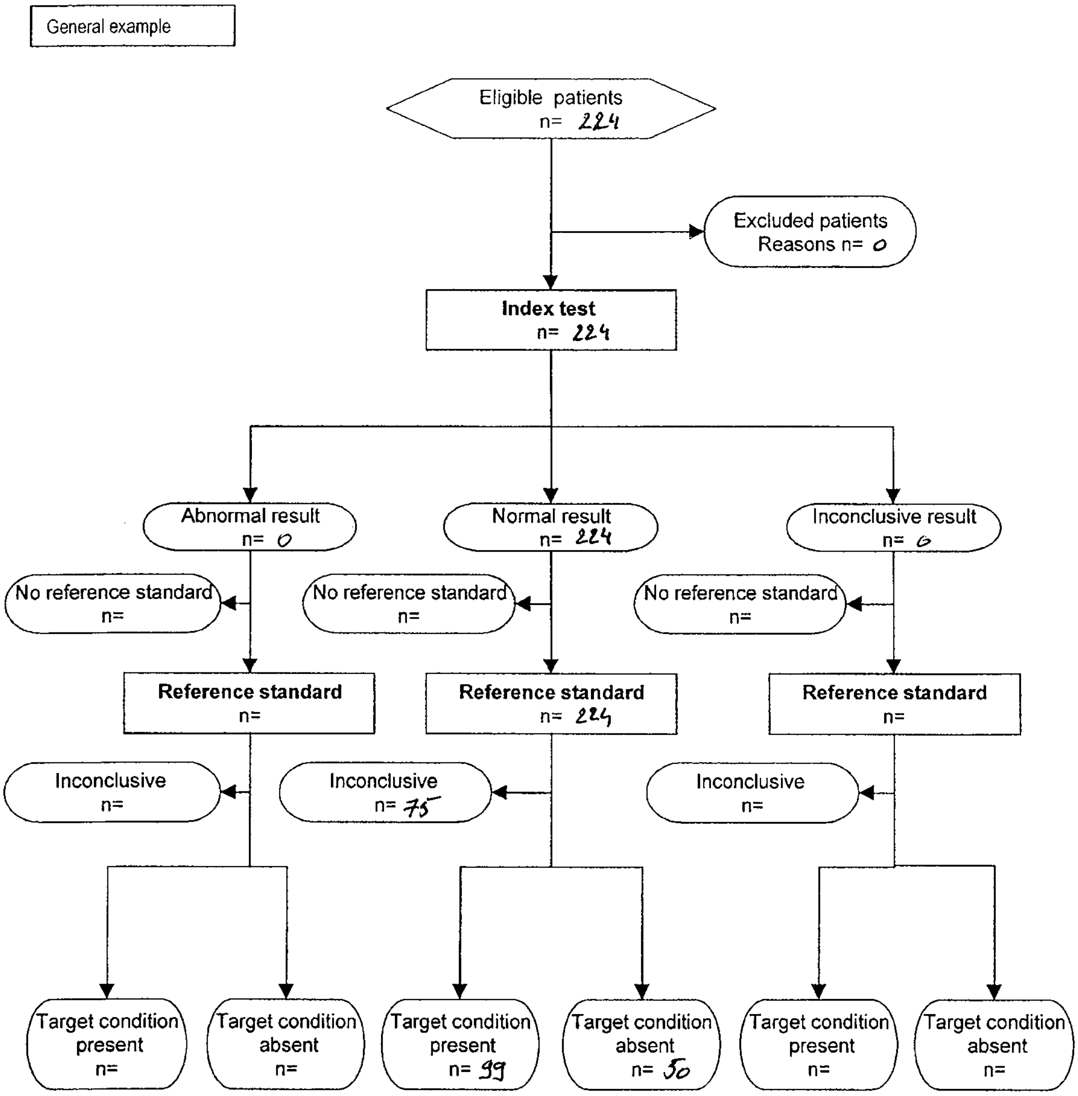

Fig. 1. Prototypical flow diagram of a diagnostic accuracy study.

reflect other designs are on the STARD Web site (see www.consort-statement.org.htm)

Discussion

The purpose of the STARD initiative is to improve the quality of the reporting of diagnostic studies. The items in the checklist and the flowchart can help authors in describing essential elements of the design and conduct of the study, the execution of tests, and the results.

We arranged the items under the usual headings of a medical research article but this is not intended to dictate the order in which they have to appear within an article.

The guiding principle in the development of the STARD checklist was to select items that would help readers to judge the potential for bias in the study and to appraise the applicability of the findings. Two other general considerations shaped the content and format of the checklist. First, the STARD group believes that one general checklist for studies of diagnostic accuracy, rather than different checklists for each field, is likely to be more widely disseminated and perhaps accepted by authors, peer reviewers, and journal editors. Although the evaluation of imaging tests differs from that of tests in the laboratory, we felt that these differences were more of degree than of kind. The second consideration was the development of a checklist specifically aimed at studies of diagnostic accuracy. We did not include general issues in the reporting of research findings, like the recommenda-
